# Low-dose post-transplant cyclophosphamide with low-dose antithymocyte globulin for prevention of graft-versus-host disease in first complete remission undergoing 10/10 HLA-matched unrelated donor peripheral blood stem cell transplants: a multicentre, randomized controlled trial

**DOI:** 10.1038/s41409-022-01754-y

**Published:** 2022-07-15

**Authors:** Yingling Zu, Zhen Li, Ruirui Gui, Yanyan Liu, Yanli Zhang, Fengkuan Yu, Huifang Zhao, Yuewen Fu, Xinrong Zhan, Zhongliang Wang, Pengtao Xing, Xianjing Wang, Huili Wang, Jian Zhou, Yongping Song

**Affiliations:** 1 Department of Hematology, Affiliated Cancer Hospital of Zhengzhou University and Henan Cancer Hospital, Zhengzhou, 450000 Henan China; 2 Academy of Medical Sciences, Zhengzhou University, Zhengzhou, 450000 Henan China; 3 Department of Hematology, Central Hospital of Xinxiang, Xinxiang, 453000 Henan China; 4 Department of Hematology, The Third People’s Hospital of Zhengzhou, Zhengzhou, 450000 Henan China; 5 Department of Hematology, the First Affiliated Hospital of Zhengzhou University, Zhengzhou, 450052 Henan China

**Keywords:** Haematopoietic stem cells, Bone marrow transplantation

## Abstract

The most widely used regimens of graft-versus-host disease (GVHD) prophylaxis in HLA-matched unrelated donor peripheral blood stem cell transplantation (MUD-PBSCT) are based on anti-thymocyte globulin (ATG) or post-transplant cyclophosphamide (PTCy). To improve the efficiency of GVHD prophylaxis, a novel regimen, composed of low-dose PTCy (20 mg/kg on day +3 and +4) and low-dose ATG (6 mg/kg), was evaluted in patients with hematological malignancies ungoing 10/10 HLA MUD-PBSCT in first remission (CR1). In our prospective, multicenter study, 104 patients were randomly assigned one-to-one to low-dose PTCy-ATG (*n* = 53) or standard-dose ATG (10 mg/kg, *n* = 51). Both the cumulative incidences (CIs) of grade II-IV acute GVHD (aGVHD) and chronic GVHD (cGVHD) at 2 years in low-dose PTCy-ATG cohort were significantly reduced (24.5% vs. 47.1%; *P* = 0.017; 14.1% vs. 33.3%; *P* = 0.013). The CI of non-relapse-mortality (NRM) was much lower (13.2% vs. 34.5%; *P* = 0.049) and GVHD-free, relapse-free survival (GRFS) was significantly improved at 2 years in low-dose PTCy-ATG arm (67.3% vs 42.3%; *P* = 0.032). The low-dose PTCy-ATG based GVHD prophylaxis is a promising strategy for patients in CR1 after 10/10 HLA MUD-PBSCT.

## Introduction

Graft-versus-host disease (GVHD) remains a major obstacle to the survival of patients after allogeneic stem cell transplantation (allo-HSCT) [1, 2]. Anti-thymocyte globulin (ATG), a conventional GVHD prophylaxis protocol, has been proven to effectively prevent acute GVHD (aGVHD) and chronic GVHD (cGVHD) [3, 4]. Hence, the updated recommendations suggest that the use of ATG has represented the standard of care of patients with matched unrelated donor (MUD) allo-HSCT in GVHD prophylaxis [5]. Nevertheless, the ATG-based regimen is associated with higher risk of aGVHD as well as infections, especially cytomegalovirus (CMV) and Epstein-Barr virus (EBV) infection [6–8]. More recently, post-transplant cyclophosphamide (PTCy) had excellent outcomes of GVHD, non-relapse mortality (NRM) and engraftment in the haploidentical HSCT (haplo-HSCT) setting [9, 10]. Since then, PTCy-based regimen had been utilized as GVHD prophylaxis in multiple clinical trials for patients with HLA-identical sibling and mismatched unrelated donor or MUD transplantation [11–15]. However, the superiority of PTCy as GVHD prophylaxis has been predominantly displayed when the allo-HSCT was performed using bone marrow (BM) as the source of stem cells [16]. Accordingly, the outcomes of of single-agent PTCy were unsatisfactory for GVHD prophylaxis in HLA-matched peripheral blood stem cell transplantation (PBSCT) [17–19].

To pursue maximum therapeutic and minimum side effects, investigators established a regimen using low-dose ATG (5 mg/kg) in conjunction with low-dose PTCy (one dose, 50 mg/kg) for GVHD prophylaxis to achieve low incidence of GVHD and potentially improve GVHD-free, relapse-free survival (GRFS) after haplo-HSCT [20, 21]. The joint use of low-dose PTCy (14.5 mg/kg on days 3 and 4) and standard-dose ATG achieved outstanding results in the haplo-HSCT setting, with significant improvments in the rates of GVHD, NRM, and GRFS [22]. In addition, the combined low-dose ATG (4.5 mg/kg) and PTCy (50 mg/kg on days 3 and 4) regimen demonstrated low rates of aGVHD and cGVHD as well as NRM, with acceptable relapse rate in MUD-PBSCT [23–25]. Up to now, the doses of ATG and PTCy in the novel regimen still remain diverse without a standard protocol. In the 10/10 HLA MUD-PBSCT, we launched a prospective, multicenter, randomized controlled clinical trial (ChiCTR2200056979) to evaluate the efficacy of low-dose ATG (6 mg/kg, Sanofi-Aventis) followed by low-dose PTCy (20 mg/kg on days 3 and 4) as GVHD prophylaxis for patients in first complete remission (CR1). The results suggested that the joint regimen had outstanding outcomes for GVHD prophylaxis in 10/10 HLA MUD-PBSCT.

## Methods

### Patients

A multicenter, randomized trial was performed in three transplant centers from March 2018 to October 2021. Patients with hematological malignancies undergoing the first 10/10 MUD-PBSCT in CR1 were eligible and randomly assigned one-to-one to two cohorts. The patients of low-dose PTCy-ATG cohort were performed with low-dose PTCy (20 mg/kg on days 3 and 4) and low-dose ATG (6 mg/kg), while the patients of standard-dose ATG cohort were administated with ATG (10 mg/kg) as GVHD prophylaxis. The study was approved by the ethical committees of each center and complied with country-specific regulatory requirements. The study was in accordance with the Declaration of Helsinki. All patients provided informed consent prior MUD-PBSC transplantation. Inclusion criteria included those patients with hematological malignancies who were eligible for MUD-HSCT. The following patients were excluded from the trial: (1) those with ECOG score >2, (2) those with active autoimmune disease, (3) those with heart, liver, or kidney dysfunction, (4) those with HIV, HBV or HCV hepatitis during active stage, (5) those with uncontrolled active bacterial and fungal infections, (6) those in pregnancy or lactation, (7) those received any other study drug within the last month.

### Transplantation procedure

All patients who underwent 10/10 MUD-HSCT received the conditioning regimen consisting of fludarabine 30 mg/m^2^/day for 5 days administered between days −6 to −2, busulfan 12.8 mg/kg administered in 16 doses between days −5 to −2, and cytosine arabinoside 2.0 g/m^2^/day for 5 days administered between days −6 to −2. Patients with lymphocytic malignancy were administered total body irradiation with 2.5~3 Gy on day −7. The protocol of PTCy 20 mg/kg on days +3 and +4, ATG 1.5 mg/kg/day on days −4 through −1, and ciclosporin (CsA) and mycophenolate (MMF) from day +5 were administrated to patients as GVHD prophylaxis in the low-dose PTCy-ATG cohort. The patients in the standard-dose ATG cohort were administered ATG 2.0 mg/kg/day on days −5 through −1 and short-course methotrexate (MTX) 10 mg/m^2^ on day +1 followed by 7 mg/m^2^ on days +3, +6, and +11, with CsA and MMF on day −1. CsA was administered at 2 mg/kg as a continuous infusion and was then tapered from day +60 without GVHD. MMF was administered at 15 mg/kg oral twice daily (maximum dose 3 g per day) and MMF tapering was started around day +30 if no aGVHD. The graft source was PBSCs mobilized with granulocyte colony stimulating factor (G-CSF).

### Supportive Care

G-CSF was given to all patients starting on day +5 at 5 µg/kg/day until absolute neutrophil count (ANC) recovery. Prophylactic ganciclovir at 5 mg/kg was given to patients in the conditioning period. Broad-spectrum antibiotics and antifungals were used for agranulocytosis or fevers. CMV-DNA in serum was routinely monitored by quantitative polymerase chain reaction twice a week until at least day +100. Preemptive therapy with ganciclovir or foscarnet was administered for CMV reactivation. EBV-DNA in whole blood was performed weekly by quantitative polymerase chain reaction. Rituximab at dose of 100 mg for adults and 50 mg/m^2^ for children was administrated on day +5 as EBV prophylaxis in the low-dose PTCy-ATG cohort.

### Engraftment, chimerism monitoring, and GVHD evaluation

Neutrophil engraftment was defined as obtaining an ANC ≥ 0.5 × 10^9^/L for three consecutive days for transplantation without G-CSF. Platelet engraftment was defined as obtaining a platelet count ≥20 × 10^9^/L for the first of seven consecutive days without platelet transfusion. Full donor chimerism was defined as ≥95% donor cells in peripheral blood and/or BM samples [26].

GVHD diagnosis was based on clinical characteristics and parenchymal biopsy. aGVHD was graded in line with the modified Glucksberg criteria [27], and cGHVD diagnosis and grades were according to the 2014 National Institutes of Health consensus criteria [28]. First-line therapy of aGVHD was methylprednisolone at 1 mg/kg/day.

### Statistical analyses

The primary end point of the study was the cumulative incidence (CI) of grade II-IV aGVHD. Secondary end points included engraftment rate, the CIs of grades III-IV aGVHD and cGVHD, the CIs of relapse (CIR) and NRM, probability of overall survival (OS), disease-free survival (DFS), and GRFS. Calculation of sample size was determined by a reduction in the CI of grade II-IV aGVHD from 46% [29, 30] in ATG-based GVHD prophylaxis to 27.9% [11] in PTCy-based GVHD prophylaxis, with a power of 80%. To detect the difference at a significance level of 5%, a total of 104 participants was required and the participants were randomly assigned to each arm. A centralized, 24-hour, internet-based randomization system was used to allocate patients into the two groups. Research staff, clinical teams, and patients were masked to randomization and treatment allocation.

NRM was defined as the time from transplant to death without relapse or progression. Relapse was defined as the time from transplant to morphologic, cytogenetic, or molecular leukemia recurrence. OS was defined as the time from transplant to death regardless of any cause. DFS was defined as survival with continuous CR after transplant. GRFS was defined as the earliest occurrence of grade III-IV aGVHD, severe cGVHD, relapse, or death from any cause after transplant.

Continuous variables and percentages for categorical variables were expressed via median values and ranges. Mann-Whitney test was used to analyze continuous variables. Differences between groups were compared with chi-square or Fisher’s exact test for categorical variables. Kaplan-Meier curves and Log-rank tests were used to estimate OS, DFS, and GRFS. A competing risk model was performed to calculate CIs, with death without relapse as a competing event for relapse, relapse or death for aGVHD, and cGVHD, with relapse as a competing risk for NRM. All *P*-values were two-sided, and *P* < 0.05 was considered statistically significant. SPSS 17.0 (Mathsoft, Seattle, WA, USA) and SAS version 9.4 (SAS Institute, Cary, NC) were used for data analyses.

## Results

### Patients

**Fig. 1:**
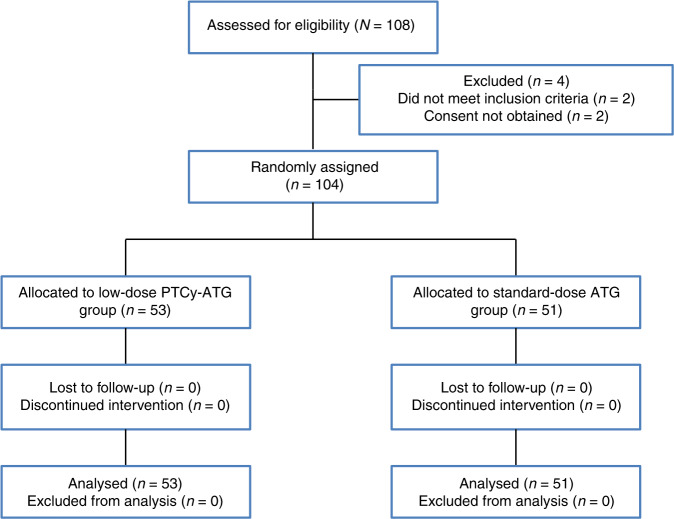
Flowchart of the study participants. PTCy post-transplant cyclophosphamide, ATG anti-thymocyte globulin.

A total of 108 patients were eligible in the study and a total of 104 patients were enrolled. 53 paitents were randomly assigned to the low-dose PTCy-ATG cohort and 51 to the standard-dose ATG cohort. Participant flow is summarized in Fig. 1. The clinical characteristics of patients and donors are summarized in Table 1. There was no significant difference with respect to patients’ age, sex, disease type, disease risk index, Kanofsky performance score, and the donors’ age or sex between the two cohorts. The median follow-up time for survivors was 561 (182–1450) days in the low-dose PTCy-ATG group as compared to 600 (196–1370) days in the standard-dose ATG group (*P* = 0.196).

**Table 1:** Patient characteristics and transplant-related parameters.

Variables	PTCy-ATG group (*N* = 53)	ATG group (*N* = 51)	*P* values
Median age in years (range)	29 (2–59)	29 (4–52)	0.969
Recipient sex			0.219
Male	28 (52.8%)	33 (64.7%)	
Female	25 (47.2%)	18 (35.3%)	
Disease type			0.362
Acute myeloid leukemia (AML)	24 (45.3%)	31 (60.8%)	
Acute lymphoblastic leukemia (ALL)	22 (41.5%)	16 (31.4%)	
Myelodysplastic syndromes (MDS)	6 (11.3%)	4 (7.8%)	
CMML	1 (1.9%)	0	
HCT-CI			0.843
≥3	8 (15.1%)	7 (13.7%)	
<3	45 (84.9%)	44 (86.3%)	
Disease risk index			0.230
Low/intermediate	26 (49.1%)	31 (60.8%)	
High/very high	27 (50.9%)	20 (39.2%)	
Minimal residual disease at transplant			0.708
Negative	30 (56.6%)	27 (52.9%)	
Positive	23 (43.4%)	24 (47.1%)	
KPS			0.563
<90	14 (26.4%)	11 (21.6%)	
≥90	39 (73.6%)	40 (78.4%)	
Donor sex			0.335
Male	48 (90.6%)	43 (84.3%)	
Female	5 (9.4%)	8 (15.7%)	
Donor age (year, median, range)	30 (21-48)	31 (18-46)	0.862
Donor-recipient pair			0.513
Female to female	2	2	
Female to male	3	6	
Male to female	23	16	
Male to male	25	27	
Blood type matching			0.723
Match	19 (35.8%)	20 (39.2%)	
Mismatch	34 (64.2%)	31 (60.8%)	
Median mononuclear cell (range, 10^8^/kg)	11.07 (1.11–25.95)	11.12 (4.43–29.30)	0.592
Median CD34 + cells (range, 10^6^/kg)	6.57 (0.16–18.69)	6.4 (1.75–35.7)	0.709
Median follow-up for survivors (range, days)	561 (182–1450)	600 (196–1370)	0.196

*PTCy* post-transplant cyclophosphamide, *ATG* anti-thymocyte globulin, *AML* acute myelocytic leukemia, *ALL* acute lymphocyte leukemia, *MDS* myelodysplastic syndrome, *CMML* chronic myelomonocytic leukemia, *HCT-CI* hematopoietic cell transplantation-comorbidity index, *KPS* Kanofsky performance score.

### Engraftment

All patients achieved engraftment in the low-dose PTCy-ATG group, while primary graft failure was observed in two patients who died of aGVHD and septic shock respectively in the standard-dose ATG group. One patient in each cohort had mixed chimerisms at day +30 (Table 2). The median numbers of mononuclear cells and CD34^+^ cells were comparable between the two cohorts. In the low-dose PTCy-ATG cohort, the median time to neutrophil recovery was one day shorter and the median time to platelet recovery was two days shorter compared to that in the standard-dose ATG cohort (12 days vs. 13 days; *P* = 0.001; and 12 days vs. 14 days; *P* = 0.002, respectively).

**Table 2:** Outcomes of two cohorts.

Variables	PTCy-ATG group (*N* = 53)	ATG group (*N* = 51)	*P* values
Time to ANC recovery (Median, days)	12 (10–15)	13 (9–19)	0.001
Time to platelets recovery	12 (9–22)	14 (9-66)	0.002
(Median, days)			
Chimerism at day +30(n, %)			
Full donor chimerism	52 (98.1)	48 (94.1)	0.289
Cumulative incidence GVHD % (95% CI)			
Grade II-IV aGVHD at day +100	24.5 (13.9–36.8)	47.1 (32.8–60.1)	0.017
Grade III-IV aGVHD at day +100	7.5 (2.4–16.7)	15.7 (7.3–27.0)	0.204
cGVHD at 2 years	14.1 (6.1–25.3)	33.3 (20.7–46.4)	0.013
Moderate/Severe cGVHD at 2 years	8.0 (2.5–17.6)	15.7 (7.3–27.0)	0.207
Cumulative incidence % (95%CI)			
Non-relapse mortality at 2 years	13.2 (5.7–23.8)	34.5 (19.7–46.0)	0.049
Relapse at 2 years	9.8 (3.5–19.9)	3.9 (0.7–12.0)	0.223
Disease-free survival at 2 years	77.0 (71.2–82.8)	61.6 (54.6–68.8)	0.217
Overall survival at 2 years	79.1 (73.5–84.7)	63.6 (56.6–70.6)	0.142
GVHD and relapse-free survival at 2 years	67.3 (60.8–73.8)	42.3 (35.2–49.4)	0.032

*ANC* absolute neutrophil count, *aGVHD* acute graft-versus-host disease, *cGVHD* chronic graft-versus-host disease, *CI* cumulative incidence.

### aGVHD and cGVHD

**Fig. 2:**
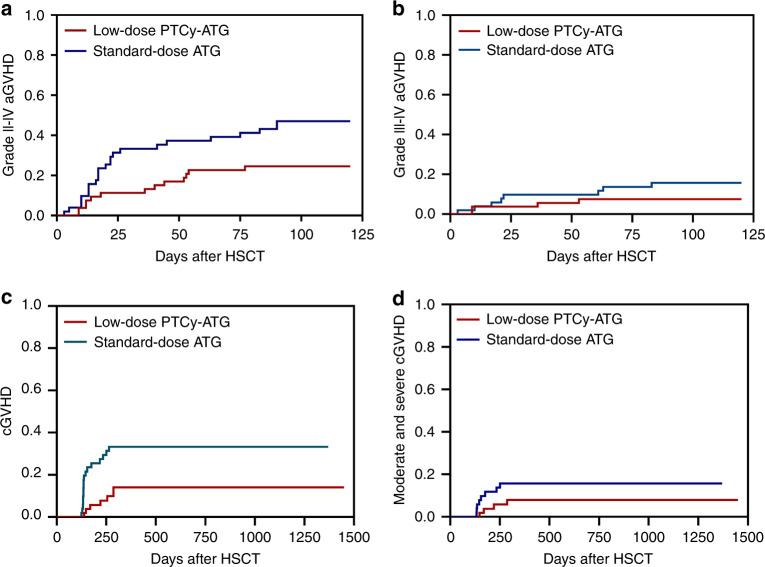
Cumulative incidences (CIs) of graft-versus-host-disease (GVHD) between low-dose post-transplant cyclophosphamide (PTCy) combined with low-dose anti-thymocyte globulin (ATG) and standard-dose ATG cohorts. **a** The CI of grade II-IV acute GVHD (aGVHD); **b** The CI of grade III-IV aGVHD; **c** The 2-year CI of chronic GVHD (cGVHD); **d** The 2-year CI of moderate to severe cGVHD.

The 100-day CI of grade II-IV aGVHD in the low-dose PTCy-ATG cohort was significantly lower as compared with that in the standard-dose ATG cohort (24.5% vs. 47.1%; *P* = 0.017) (Table 2) (Fig. 2a). There was no significant difference in CI of grade III-IV aGVHD between the two groups (7.5% vs. 15.7%; *P* = 0.204) (Fig. 2b). No patient suffered from late onset aGVHD, so the CIs of aGVHD at day +100 and +180 were the same. The 2-year CI of cGVHD was significantly lower as 14.1% in low-dose ATG-PTCy group as compared to 33.3% in the standard-dose ATG group (*P* = 0.013) (Fig. 2c). However, the rates of moderate to severe cGVHD at 2 years were comparable between two cohorts (8.0% vs. 15.7%; *P* = 0.207) (Fig. 2d).

### Infection complications

As listed in Table 3, the 100-day incidences of CMV reactivation and CMV disease were comparable between the two cohorts (50.9% vs. 47.1%; *P* = 0.692; and 3.8% vs. 2.0%; *P* = 0.581, respectively). The median time to CMV reactivation was +46 days (range 23 to 82) and +36 days (range 19 to 87) in the low-dose PTCy-ATG and standard-dose ATG cohorts, respectively. There was no significant difference in the time of CMV reactivation between the two groups (*P* = 0.212). The 2-year incidence of EBV reactivation in low-dose PTCy-ATG cohort was significantly lower (15.1% vs. 60.8%; *P* = 0.000). The 2-year incidence of post-transplantation lymphoproliferative disorder (PTLD) between the two cohorts was comparable (0% vs. 2.0%; *P* = 0.490). The incidences of hemorrhagic cystitis and pulmonary infection were significantly lower in low-dose PTCy-ATG cohort as compared to those in standard-dose ATG cohort (37.7% vs.62.7%; *P* = 0.011; and 35.8% vs. 58.8%; *P* = 0.019, respectively).

**Table 3:** Complications of two cohorts.

Complications (*n*, %)	PTCy-ATG group (*N* = 53)	ATG group (*N* = 51)	*P* values
Pulmonary infection	19 (35.8)	30 (58.8)	0.019
CMV	27 (50.9)	24 (47.1)	0.692
CMV disease	2 (3.8)	1 (2.0)	0.581
EBV	8 (15.1)	31(60.8)	0.000
PTLD	0 (0.0)	1 (2.0)	0.490
Hemorrhagic cystitis	20 (37.7)	32 (62.7)	0.011

*CMV* cytomegalovirus, *EBV* Epstein-Barr virus, *PTLD* posttransplantation lymphoproliferative disorders.

### Outcomes

**Fig. 3:**
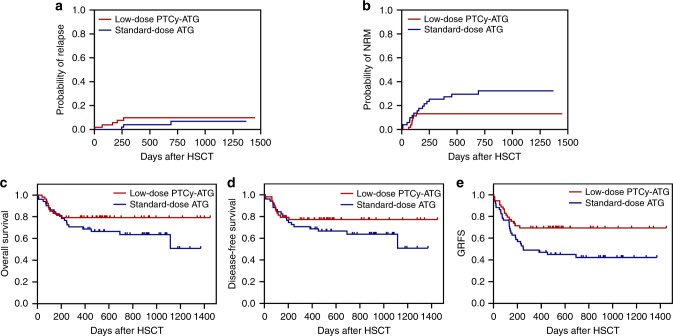
Clinical outcomes after matched unrelated donor peripheral blood stem cell transplantation (MUD-PBSCT) between low-dose post-transplant cyclophosphamide (PTCy) combined with low-dose anti-thymocyte globulin (ATG) and standard-dose ATG cohorts. **a** The 2-year CI of relapse; **b** The 2-year CI of non-relapse-mortality (NRM); **c** The 2-year probability of overall survival (OS); **d** The 2-year probability of disease-free survival (DFS); **e** The 2-year CI of GVHD-free, relapse-free survival (GRFS).

The median follow-up time was similar among the two cohorts. The 2-year CIR were similar among the two cohorts (9.8% vs. 3.9%; *P* = 0.223) (Fig. 3a). The median time to relapse was 4.5 months (range 4–6 months) in the low-dose PTCy-ATG cohort and 5 months (range 3–23 months) in the standard-dose ATG cohort. The 2-year CI of NRM in cohort low-dose PTCy-ATG was significantly reduced (13.2% vs. 34.5%; *P* = 0.049) (Fig. 3b). The 2-year probabilities of OS and DFS were comparable between the two cohorts (79.1% vs. 63.6%; *P* = 0.142; 77.0% vs. 61.6%; *P* = 0.236, respectively) (Fig. 3c, d). The 2-year probability of GRFS in the low-dose PTCy cohort was significantly improved (67.3% vs. 42.3%; *P* = 0.032) (Fig. 3e).

The causes of death are shown in Table 4. Relapse was the most common cause of death in the low-dose PTCy-ATG cohort, while infection was the foremost reason of death in the standard-dose ATG cohort.

**Table 4:** Causes of death.

Cause of death	PTCy-ATG group (*N* = 12)	ATG group (*N* = 19)
Infection other than CMV/EBV	4 (33.3%)	7 (36.8%)
Relapse	5 (41.7%)	3 (15.8%)
GVHD	1 (8.3%)	3 (15.8%)
Organ failure	1 (8.3%)	4 (21.1%)
TMA	1 (8.3%)	2 (10.5%)

*CMV* cytomegalovirus, *EBV* Epstein-Barr virus, *GVHD* graft-versus-host disease, *TMA* thrombotic microangiopathy.

## Discussion

Data from the prospective study showed that the CIs of grade II-IV aGVHD and cGVHD were significantly lower in the low-dose PTCy-ATG cohort as compared with the standard-dose ATG cohort. Meanwhile, lower NRM and improved GRFS have also been achieved for patients with low-dose PTCy-ATG. Our observations highlighted that the novel regimen consisting of low-dose PTCy and low-dose ATG in GVHD prophylaxis had promising activity and improved outcomes of patients in CR1 after 10/10 MUD-PBSCT.

Over recent years, GVHD prophylaxis is focused on the use of ATG and PTCy. ATG-based regimens demonstrated the capability of aiding reliable engraftment and alleviating GVHD. However, there are increased risks of infection and relapse as well as delayed immunological recovery [31, 32]. PTCy has been preferred on inducing the apoptosis of early alloreactive T-cells after transplant and reducing the risks of GVHD and graft rejection, while enhancing potential antineoplastic activity [16, 33, 34]. Nevertheless, prolonged time to engraftment and immune reconstitution were accompanied by the use of high-dose PTCy, which relied on the dosage of PTCy [35, 36]. However, some studies reported that PTCy-based regimens for GVHD prophylaxis in MUD-PBSCT were associated with relative high incidence of grade II-IV aGVHD (28%–59%) [11, 37–39]. With the aim to lower the risk of GVHD and improve outcomes, investigators set out to study the regimen of PTCy in conjunction with ATG in MUD-PBSCT. In a previous study with unrelated HSCT donors, the results revealed that CIs of aGVHD and grade III–IV aGVHD in PTCy-ATG cohort (PTCy 50 mg/kg on days +3 and +4 combined with ATG 4.5 mg/kg) were significantly lower than those in ATG cohort (17% vs.33%; *P* = 0.084; anf 7% vs.25%; *P* = 0.0395, respectively) [25]. Prem et al. [23] reported the results with PTCy (50 mg/kg on days +3 and +4) combined with ATG (4.5 mg/kg) as GVHD prophylaxis, the incidences of grade II-IV and grade III-IV aGVHD were 31.6% and 11.8%, and the rate of cGVHD was 21% in MUD-PBSCT. In our study, the joint regimen could lower the CIs of grade II-IV aGVHD and cGVHD, which revealed that low-dose PTCy-ATG could be a promising regimen for GVHD prophylaxis after MUD-PBSCT. When PBSCs were used as the graft, the risks of grade II–IV aGVHD and cGVHD increased in recipients of haplo-HSCT [40, 41]. The infusion of low-dose ATG could deplete early active T lymphocytes, while the administration of low-dose PTCy on days +3 and +4 could eradicate rapidly proliferating T cells [10]. The synergistic effects due to different action mechanisms of ATG and PTCy on T lymphocyte depletion devoted to reduce risk of GVHD. In additon, grade III-IV aGVHD and moderate to severe cGVHD were comparable between two cohorts in present study even though wide numerical differences, explained by samll sample size. Hence, larger sample sizes are required to futher evaluate the efficacy of the join regimen.

Previous studies have observed prolonged time to engraftment due to the administration of PTCy, even delaying the time to neutrophil engraftment as long as a week [36, 37]. However, inconsistent with prior studies, the median implantation time of neutrophils and platelets was significantly shorter as compared to that with standard-dose ATG regimen, which might explained that the addition of low-dose ATG at pre-transplantation would accelerated the hematopoietic reconstitution. Furthermore, the administration of MTX based on cytotoxic effect in standard-dose cohort might contribute to prolonged time to engraftment. Previous studies also demonstrated that low/high-dose PTCy combined with ATG is effective in alleviating GVHD without impact on relapse [22, 42], and PTCy has been displayed to be capable of separate GVHD and a graft-versus-leukemia effect in preclinical experiments [34]. Consistent with previous studies, CIRs was comparable among two cohorts, even though the patients with MRD at transplant accounted for high proportion, might be explained by the administration of cytosine arabinoside in the conditioning regimen, and the research with larger samples may yield rigorous results in view of numerical difference in relapse. As for survival outcomes, aGVHD and cGVHD are the largest contributor to NRM after HSCT. The 2-year probability of NRM of low-dose PTCy-ATG regimen in our study was 13.2%, which was relatively lower as compared to the results of PTCy-based regimen (16%) or ATG-based regimens (36%) [37]. Furthermore, 1-year probability of NRM was 9.2–21.1% in recent reports for patients with low-dose ATG and PTCy-based GVHD prophylaxis regimens after MUD-HSCT [23, 43]. In our study, low-dose PTCy‐ATG platform was found to yield better 2-year GRFS rate (67.3%), while the incidence of GRFS was only 44–52% for patients with PTCy-based regimen, ATG-based regimen or PTCy-ATG regimens in the MUD-HSCT [23, 37, 44]. It is speculated that the low-dose PTCy-ATG baesd prophylaxis represents a promising strategy for alleviating GVHD and improving survival.

The joint use of PTCy and ATG was associated to increased infection, explained by the dual immunosuppression of PTCy and ATG. The rates of CMV reactivation among the two cohorts were similar, and the observed rate was comparable with previous studies on the combination of high-dose PTCy and low-dose ATG (49.0%) [23]. In present study, the incidences of pulmonary infection and hemorrhagic cystitis were lower in the low-dose PTCy-ATG cohort. We speculated that immunosuppression was relatively weak due to the use of low dose of ATG. As for EBV reactivation, the rate obtained in our study (15.1%) was remarkably lower when compared with standard-dose ATG regimen (60.8%) and another study that adopted low-dose PTCy along with ATG (21.0%) [22]. The novel strategy of rituximab on day +5 may contribute to the low rate of EBV reactivation, apart from the fact that PTCy resulted in decreased or absence of incidence of PTLD [45]. Bacigalupo et al. reported for the first time that a dosage of rituximab (200 mg) on day +5 led to a decreased incidences of EBV reactivation and aGVHD without adding infectious episodes for alternative donor HSCT [46]. In our study, the dose of rituximab was fixed at 100 mg for adult and 50 mg/m^2^ for children, which had a similar effect in terms of EBV prophylaxis. Nevertheless, the use of rituximab only to low-dose PTCy-ATG arm was the limitation of the study, and the follow-up experiments will not repeat the administration of rituximab to address the concern.

Moreover, the dose of ATG was what we needed pay attention. The standard-dose of ATG was based on the Beijing protocol comprising T-cell depletion with high-dsoe ATG and strengthened immune suppression, but accompanied with high risk of infection. Hence, the novel regimen was administrated to evaluate the impact on GVHD and infection after MUD-HSCT. Seo et al.’s report [47] showed that with ATG 7.5 mg/kg for GVHD prophylaxis, grade II-IV, and grade III-IV aGVHD were 20.0% and 20.0% in absolute lymphocyte count (ALC) < 500/ul group, while were 32.7% and 16.3% in ALC ⩾500/μl group. In the cause of death, infection accounted for 70.0% and 41.2% in two group, respectively. The addition of 4.5 mg/kg ATG to a backbone of PTCy, the rates of grade II-IV and grade III-IV aGVHD were 6.2%-20.1% and 4.6%, the proportion of death caused by infection was 22.2–41.2% in the MUD-HSCT setting [24, 43]. In present study, the CIs of grade II-IV and grade III-IV aGVHD were 24.5% and 7.5%, and death due to infection accounted for 33.3% in the PTCy-ATG cohort. Our data in corporation with these results suggested that low-dose ATG and PTCy might not increase the risk of infection. Nevertheless, the optimal dose of ATG in the combined regimen remains to be further explored with randomized and controlled trials on the basis of long-term follow-up and large samples.

This study had several limitations, including between-group difference of rituximab, lower number of samples, shorter follow-up, etc. Further studies with high methodological quality are needed to verify the feasibility of the strategy in the future. Inter-institutional comparison is another limitation. Although the identical protocol is adopted in all three centers and standard of transplantation is really well matched, we cannot completely rule out the existence of small differences in medical practices, which may influence transplant outcomes to some extent. In conclusion, the results of our study displayed decreased risk of GVHD as well as excellent engraftment and disease control. The low-dose PTCy-ATG based GVHD prophylaxis might be a promising protocol for patients undergoing MUD-PBSCT in CR1.

## Data Availability

The datasets used and/or analyzed during the current study are available from the corresponding author on reasonable request.
